# Protective role of ceftriaxone plus sulbactam with VRP1034 on oxidative stress, hematological and enzymatic parameters in cadmium toxicity induced rat model

**DOI:** 10.2478/v10102-012-0032-3

**Published:** 2012-12

**Authors:** Vivek Kumar Dwivedi, Anuj Bhatanagar, Manu Chaudhary

**Affiliations:** 1 Preclinical Divison, Venus Medicine Research Centre, Baddi, H.P. 173205 India; 2 Analytical Division, Venus Medicine Research Centre, Baddi, H.P. 173205 India

**Keywords:** cadmium toxicity, hematological and biochemical parameters, oxidative stress and enzymatic parameters, hepatic and renal tissues, Elores

## Abstract

We investigated the protective role of ceftriaxone plus sulbactam with VRP1034 (Elores) on hematological, lipid peroxidation, antioxidant enzymatic activities and Cd levels in the blood and tissues of cadmium exposed rats. Twenty-four male rats were divided into three groups of eight rats each. The control group received distilled water whereas group II received CdCl_2_ (1.5 mg/4 ml/body weight) through gastric gavage for 21 days. Group III received CdCl_2_ and was treated with ceftriaxone plus sulbactam with VRP1034 for 21 days. The hematological, biochemical, lipid peroxidation levels and enzymatic parameters were measured in plasma and tissues (brain, liver and kidney) of all groups. The Cd, Zn and Fe levels were measured in blood and tissues of all groups. Our findings showed significantly decreased cadmium (*p<*0.001), malonaldialdehyde (*p<*0.001) and myloperoxidase (MPO) levels along with significantly increased hemoglobin (*p<*0.01), RBC (*p<*0.05), hematocrit (*p<*0.05) levels and all antioxidant enzymatic activities (SOD, CAT, GR, GPx) in plasma and tissues of ceftriaxone plus sulbactam with VRP1034 treated group as compared to cadmium exposed group. Delta aminolevulinate dehydratase (δ-ALAD) activity was significantly (*p<*0.001) increased in the blood of ceftriaxone plus sulbactam with VRP1034 treated group as compared with cadmium exposed group. The levels of hepatic and renal parameters were significantly (*p<*0.001) decreased in ceftriaxone plus sulbactam with VRP1034 treated group as compared to cadmium exposed group. These findings indicate that ceftriaxone plus sulbactam with VRP1034 acts as a potent free radical scavenger and exhibits metal chelating properties that reduce free radical mediated tissue injury and prevent dysfunction of hepatic and renal organs during metal intoxication.

**LIST OF ABBREVIATIONS**
SOD: superoxide dismutase
CAT: catalase
GR: glutathione reductase
Gpx: gluathione peroxidase
GST: Gluathione S-transferase
MDA: malon dialdehyde
GSH: reduced glutahione
GSSG: Oxidized glutathione
MPO: myloperoxidase
FDC: fixed dose combination
XO: Xanthine oxidase

## Introduction

Cadmium (Cd) is an industrial and environmental pollutant, arising primarily from batteries, electroplating, pigment, plastic, fertilizer industries, and cigarette smoke. It is dangerous because humans consume both plants and animals that absorb Cd efficiently and concentrate it within their tissues (Stohs & Bagchi, 1995) . Cd operates by various mechanisms of toxicity in particular species and under different experimental conditions (Iscan *et al.*, 1994; Žikic *et al.*, 1996; Waisberg *et al.*, 2003). Cd was found to stimulate free radical production, resulting in oxidative deterioration of lipids, proteins and DNA, and initiating various pathological conditions in humans and animals (Waisberg *et al.*, 2003). Chronic exposure to inorganic Cd results in accumulation of the metal mainly in the liver and kidneys, as well as in other tissues and organs, causing many metabolic and histological changes, membrane damage, altered gene expression and apoptosis (Waisberg *et al.*, 2003; Shaikh *et al.*, 1999; Casalino *et al.*, 2002a). A number of cadmium-induced effects include deterioration of cell to cell adhesion, DNA-related processes cell signaling and energy metabolism, implying that this metal acts on different molecular targets in human organs. Cadmium was shown to induce apoptosis in mouse liver (Michael *et al.,* 1999).

Ceftriaxone plus sulbactam with VRP1034 is a novel fixed dose combination drug. Ceftriaxone is the third generation the cephalosporin class of antibiotics, whereas sulbactam is a β-lacatamase inhibitor. It is well established that cephalosporins act as multidentate chelating agents (Anacona & Rodriguez, 2005; Anacona & Osorio, 2008). Besides this property, cephalosporins are known as a thioether containing class of antibiotics, which are rather effective in preventing free radical-mediated oxidation of sulfhydryl groups in the antibiotics. Various studies reported that ceftriaxone and sulbactam individually showed free radical scavenging property (Lapenna *et al.*, 1995; Gunther et al, 1993). It is well known that sulbactam acts as oxidant scavenger and inhibits *in vitro* neutrophil function (Santos and Arbo, 1989). VRP1034 is a third vector between these two drugs. VRP1034 has chelating and free radical scavenger properties. VRP1034 is a patent protected trade secrete. It has already been reported that a novel fixed dose combination of ceftriaxone plus sulbactam with VRP1034 play also a significant role in various bacterial infections (Dwivedi *et al.,* 2010). The aim of this study was to investigate the protective effect of ceftriaxone plus sulbactam with VRP1034 on hematological, biochemical and some enzymatic parameters in blood and tissues of cadmium exposed rats.

## Material and methods

### Chemicals

All biochemicals used in the present study were procured from Sigma, St. Louis, MO, USA. δ-aminolevulinic acid (δ-ALA) and cadmium chloride were purchased from Sigma Chemical, St Louis, MO, USA. Other chemicals purchased locally, were of analytical grade. Ketamine hydrochloride was purchased from Samarth Life Science Pvt. Ltd. Mumbai. Other biochemical kits were procured from Erba Diagnostics Mannheim Gmb, Germanny.

### Drugs

Ceftriaxone plus sulbactam with VRP1034 was obtained from Venus Remedies Ltd., Baddi, H.P. The ratio of ceftriaxone plus sulbactam to VRP1034 was 2:1 respectively.

### Animals

The animals were obtained from the animal house facility of Venus Medicine Research Centre, Baddi, H.P. The experiment was carried out after approval from the Institutional Animal Ethics Committee (IAEC). The IAEC number for this study was IAEC/CSV/2011-05. The study was performed on male wistar rats weighing 140±10 g, housed in polypropylene cages in an air-conditioned room with temperature maintained at 25±2 °C and 12 h alternating day and night cycles. The animals were allowed standard rat chow diet and sterile distilled water.

### Experimental design

Twenty four male rats were selected and divided into three groups of eight rat each:

- Group I: Control normal saline treated group
- Group II: CdCl_2_ induced group (1.5mg /4ml /kg body weight)
- Group III:CdCl_2_+Ceftriaxone plus sulbactam with VRP1034 treated group (155.0 mg/kg body weight/day)

Cadmium chloride (CdCl_2_) was given to sixteen animals through gastric gavage daily for 21 days and eight animals received plane distilled water and served as control. Toxicity was confirmed after showing signs such as loss of appetite, body weight loss, decreased hemoglobin and increased body temperature. After confirmation of toxicity, drug was given only to group III animals through intravenous route for 21 days and body weight, body temperature, food and water intake along with hematological parameters were recorded. All the animals were decapitated 24 hour after last treatment, 2.5 ml blood was collected in EDTA containing vials and liver, kidney and brain tissues were collected in chilled phosphate buffer saline and washed three times with chilled PBS and homogenates were prepared for measurement of biochemical and enzymatic parameters.

### Plasma preparation

A sample of 1.5 ml blood was centrifuged at 6 000 rpm for 15 minutes and the supernatant was carefully taken into a polypropylene tube and stored at 2–8 °C for measurement of antioxidant enzymatic and biochemical parameters. The remaining part of blood samples was used for determination of metal and δ-aminolevulinic acid dehydratase activity.

### Homogenate preparation

Liver, brain and kidney tissue homogenates (10%) were prepared in chilled phosphate buffer-NaCl solution containing 0.15 mol/L NaCl in 0.05 mol/L, Na_2_HPO_4_-NaH_2_PO_4_ buffer (pH 7.2) and left for at least 1 hr at 2–8 °C before measurement of enzymatic and biochemical parameters.

### Determination of hematological parameters

Hematological parameters were tested with automatic cell counter (Sysmex XT 2000i).

### Blood δ-aminolevulinic acid dehydratase (ALAD) activity

ALAD activity was assayed in the blood according to the method of Berlin and Schaller (1974). For measurment of δ-ALAD activity, 0.2ml of blood was taken and mixed with 1.3ml double distilled water and the test tubes were incubated for 10 minutes at 37°C for complete hemolysis. After incubation of all test tubes, 1.0 ml of δ-ALA standard solution was added and further all test tubes were incubated for one hr at 37 °C. The reaction was stopped after one hr by adding 1.0 ml of 10% TCA solution. All test tubes were centrifuged at 600 g for 5 to 10 min. After centrifugation, 1.5 ml of supernatant was taken in clean test tubes and equal amount of Ehrlich reagent was added and absorbance was recorded at 555 nm wavelength after 5 min. The molar extension coefficient 6.1 × 10^4^ was used for calculation.

### Enzymatic parameters

All enzymatic parameters were standardized at 25 °C

### Superoxide dismutase assay (SOD, EC 1.15.1.1)

SOD activity was determined in plasma and tissues by the Method of Misra and Fradovich (1972). The reaction mixture consisted of 1.0 ml carbonate buffer (0.2 M, pH 10.2), 0.8 ml KCl (0.015M), 0.1 ml of plasma and tissue and water to reach the final volume of 3.0 ml. The reaction was started by adding 0.2 ml of epinephrine (0.025M). The change in absorbance was recorded at 480 nm at 15 second intervals for one minute at 25 °C. Suitable control lacking enzyme preparation was run simultaneously.

One unit of enzyme activity is defined as the amount of enzyme causing 50% inhibition of auto-oxidation of epinephrine.

### Catalase assay (CAT; EC.1.11.1.6 )

Catalase activity was measured in plasma and tissues according to the procedure of Aebi (1984) at room temperature with slight modification. Plasma (100 µl) and 0.025 of ml tissues were added in clean tubes and kept on ice bath for 30 minutes at room temperature. Triton-X (10 µl) was added to each plasma and tissue containing test tube. In a cuvette, to 200 µl phosphate buffer (0.2 M; pH 6.8) , 20 µl of sample and 2.53 ml distilled water was added. The reaction was started by adding 250 µl of H_2_O_2_ (0.066 M in phosphate buffer) and decrease in optical density was recorded at 240 nm wave length every 15 second for one minute. The molar extinction coefficient of 43.6 M Cm^－1^ was used for determination of catalase activity.

One Unit of enzyme activity was defined as the amount of enzyme that liberates half of the peroxide oxygen from H_2_O_2_ in one minute at 25 °C.

### Glutathione reductase activity (GR; EC.1.6.4.2)

GR activity was measured in plasma and tissues by the method of Carlberg and Mannervik (1985). The reaction mixture consisted of 1.5 ml potassium phosphate buffer (0.2 M, pH 7.0) containing 2 mM EDTA, 0.15 ml of 2 mM NAPDH , 0.2 ml of 20 mM oxidized glutathione and distilled water to make up the final volume of 3.0 ml. The reaction was started by adding 0.1 ml of plasma homogenate in the linearity range. The absorbance was measured at 340 nm for one minute at 15 sec. intervals. Control lacking enzyme was run simultaneously.

One unit of GR activity is expressed as the amount of NADP formed in one minute by one ml of enzyme preparation. Calculation of the enzyme activity was done by using the molar extinction coefficient of NADPH as 6.22 × 10^3^.

### Glutathione peroxidase activity (GPx; EC I .ll. 1.9)

GPx activity was measured by the method described by Rotruck *et al.,* (1973). The reaction mixture contained 0.2 ml of 0.4 M Tris-HCl buffer pH 7.0, 0.1 ml of 10 mM sodium azide, 0.2 ml of homogenate, 0.2 ml glutathione, 0.1 ml of 0.2 mM H_2_O_2._The contents were incubated at 37 °C for 10 min. The reaction was arrested by 0.4 ml of 10% TCA and centrifuged. The supernatant was assayed for glutathione content by using Ellmans reagent (19.8 mg of 5,5’-dithiobisnitro benzoic acid (DTNB) in 100 ml of 0.1% sodium nitrate).

### Glutathione S-transferase (GST; EC 2.5.1.18) activity

Glutathione S-transferase activity in plasma and tissue spectrophotometry at 480 nm and 37 °C was followed by conjugation of the acceptor substrate1-chloro-2, 4-dinitrobenzen as described by Herbig *et al.* (1974). The absorbance was calculated from the extinction coefficient 9.6 mM/Cm.

### Reduced glutathione (GSH) measurement

Reduced glutathione was assayed by the method of Ellman (1959). Samples of 0.5 ml plasma and 0.25 ml tissue were mixed with equal amount of 5% (w/v) TCA reagent and kept for 10 min at room temperature, proteins were precipitated and the filterate was removed carefully after centrifugation at 3 500 rpm for 15 minutes. Filtrate (0.25 ml) was taken and added to 2.0 ml of Na_2_HPO_4_ (4.25%)and 0.04 ml of DTNB (0.04%). A blank sample was prepared in similar manner using double distilled water instead of the filtrate. A pale yellow color was developed and optical density was measured at 412 nm by a spectrophotometer.

### Total thiol determination

Total thiol content was analyzed by the method of Hu (1994). Plasma and tissue samples (0.2 ml) were taken in test tubes and 0.6 ml of Tris EDTA buffer (Tris 0.25 M; EDTA 20mM; pH 8.2) was added, followed by 40 µl of 10 mM of 5,5’ dithionitrobis 2-nitrobenzoic acid (DTNB in methanol) and the total reaction volume of 4.0 ml was obtained by adding 3.16 ml of methanol. All test tubes were sealed and the color was developed for 15–20 min, followed by centrifugation at 3 000 g for 10–15 min at room temperature. The absorbance of the supernatant was measured at 412 nm wavelength.

### Xanthine oxidase (XO; EC 1.17.3.2 ) activity

XO (Xanthine oxidase) was assayed in plasma and homogenate by the method of Fried and Fried (1945). The reaction mixture consisted of 0.9 ml of 0.1 M phosphate buffer pH 7.8; 0.75 ml 10 mM EDTA; 0.15 ml 0.2 mg/ml phenazine methosulphate; 0.45 ml 4 mg/ml nitroblue tetrazolium (NBT) salt; 0.5 ml; 1 mM xanthine and water to reach the volume of 3.5 ml. The reaction was started at 37 °C by addition of 0.5 ml of 1 mM Xanthine for 1 min. Extinction coefficient of the reduced NBT at 540 nm is 7.2 cm/µmole. One unit of enzyme activity was defined as the amount of enzyme that converted 1 mole of xanthine to uric acid in one minute at specified assay conditions.

### Measurement of myleoperoxidase (MPO; EC 1.11.2.2)

Myeloperoxidase was determined by O-dianisidine method with slight modification Kurutas *et al.,* (2005). The assay mixture consisted of 0.3 ml of sodium phosphate buffer (0.1 M; pH 6.0), 0.3 mL of H_2_O_2_ (0.01 M), 0.2 mL of O-dianisidine (0.02 M) (freshly prepared in distilled water) and final volume of 3.0 ml was obtained with distilled water. The reaction was started by addition of 0.025 ml samples. The change in absorbance was recorded at 460 nm wavelength. All measurements were carried out in duplicate. One unit of enzyme activity was defined as the increase in absorbance of 0.001 min^–1^.

### Measurement of lipid peroxidation level

Free radical mediated damage was assessed by the measurement of lipid peroxidation in the term of malon dialdehyde (MDA) formed, essentially according to method of Ohkawa *et al.*, (1979). It was determined by thio barbituric reaction. The reaction mixture consisted of 0.2 ml samples, 0.20 ml of 8.1% sodium dodecyl sulphate (SDS), 1.5 ml of (20%, pH 3.5) acetic acid, 1.5 ml of 0.8% thio barbituric acid (TBA) and 0.6 ml distilled water to see find volume of 4.0 ml. The tubes were kept in boiled water at 95^0^C for one hr and cooled immediately under running tap water. The amount of 1.0 ml of water was added to and 5.0 ml of the mixture of n-butanol and pyridine (15:1 v/v) and vortexed. The tubes were centrifuged at 3500 rpm for 15–20 minutes. The upper layer was aspirated and optical density was measured at 532 nm. The molar extension coefficient 1.56x 10^5^ was used for calculation.

### Determination of biochemical parameters

The total protein, hepatic and renal enzymes (SGOT, SGPT, Creatinine and ALP levels) were measured in the plasma sample by standard kit method.

### Metal determination

For assessment of cadmium, iron and zinc concentration in blood and tissues, 0.5 ml samples (blood, liver, kidney and brain) were mixed with 4.5 ml of acidic glycerol (1% HNO_3_ and 5% glycerol mixture) and the final volume of 10.0 ml was obtained with distilled water. Cadmium, iron and zinc were measured by using a flame atomic absorption spectrophotometer (Analytikjena, model No. contra A300, Germany) with hollow-cathode lamp at wave length 228.8 nm (Cd), 248.0 nm (Fe) and 213.9 nm (Zn). The direct absorption of the solution was determined by the atomic absorption spectrophotometer and suitable standard curves of each metal were prepared by using 20 to 100 µg/ml. All chemicals used for metal measurements were of Merck reagent grade.

### Statistical analysis

All values were expressed as Mean ± SD. One-way analysis of variance (ANOVA) followed by Newman-Keuls comparison test was used to determine statistical difference between control vs cadmium exposed group and cadmium exposed group vs ceftriaxone plus sulbactam with VRP1034 treated group. The *p*-values <0.05 were considered statistically significant.

## Results

**Figure 1:**
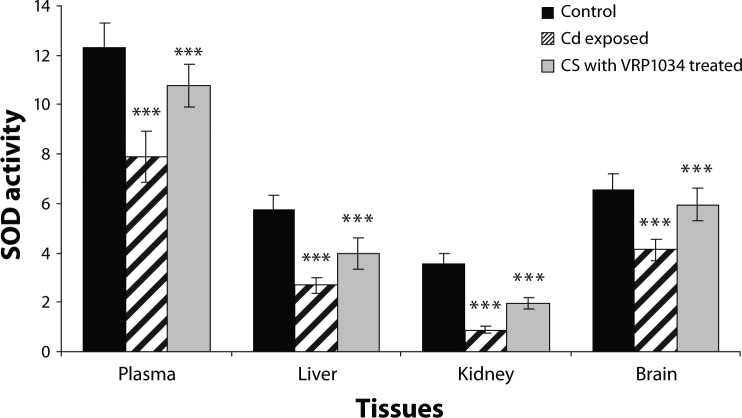
Effect of Ceftriaxone plus sulbactam with VRP1034 on Superoxide dismutase activity in cadmium exposed rat. All data are Mean ± SD of each group. Activity expressed in plasma (µmol/min/ml) whereas in liver, kidney and brain tissue (µmol/min/g tissue). Data are compared between control *vs* Cd exposed group and Cd exposed *vs* C+S with VRP1034 treated group. Where ***p<0.001 (highly significant); **p<0.01 (significant); *p<0.05 (significant) and ^Ns^ *p>*0.05.

**Figure 2:**
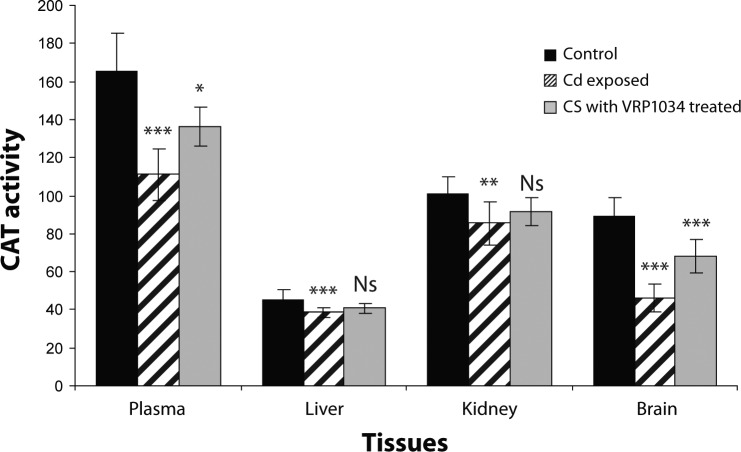
Effect of ceftriaxone plus sulbactam with VRP1034 on Catalase activity in cadmium exposed rat. All data are Mean ± SD of eight animal each group. Catalase activity was expressed in plasma (µmol/min/ml) whereas in liver, kidney and brain tissues (µmol/min/g tissue). Data are compared between control *vs* Cd exposed group and Cd exposed *vs* C+S with VRP1034 treated group. Where ***p<0.001 (highly significant); **p<0.01 (significant); *p<0.05 (significant) and ^Ns^ *p>*0.05.

**Figure 3:**
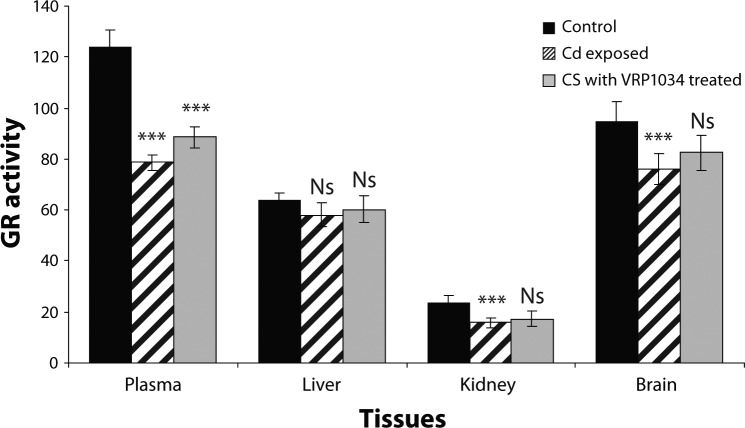
Effect of ceftriaxone plus sulbactam with VRP1034 on glutathione reductase (GR) activity in cadmium exposed rat. All data are Mean ± SD of each group. Activity expressed in plasma (µmol/min/ml) whereas in liver, kidney and brain tissue (µmol/min/g tissue). Data are compared between control *vs* Cd exposed group and Cd exposed *vs* C+S with VRP1034 treated group. Where ***p<0.001 (highly significant); **p<0.01 (significant); *p<0.05 (significant) and ^Ns^ *p>*0.05.

**Figure 4:**
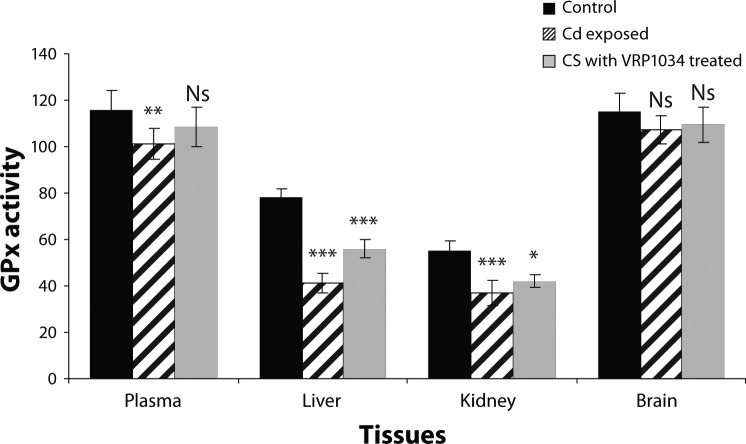
Effect of Sulbactomax on glutathione peroxidase (GPx) activity in cadmium exposed rat. All data are Mean ± SD. GPx activity expressed in plasma (µmol/min/ml) whereas in liver, kidney and brain tissue (µmol/min/g tissue). Data are compared between control *vs* Cd exposed group and Cd exposed *vs* C+S with VRP1034 treated group. Where ***p<0.001 (highly significant); **p<0.01 (significant); *p<0.05 (significant) and ^Ns^ *p>*0.05.

SOD and CAT enzyme activities were significantly decreased (*p<*0.001) in plasma, (*p<*0.001) in brain, (*p<*0.001; *p<*0.01), in liver ( *p<*0.001) and (*p<*0.001; *p<*0.01) in kidney tissues of Cd exposed group as compared to control group. After treatment with ceftriaxone plus sulbactam with VRP1034 for three weeks, these enzyme activities were significantly increased (*p<*0.01; *p<*0.05) in plasma and (*p<*0.001) in brain tissues of the treated group as compared to the Cd exposed group. In liver and kidney tissues only SOD activity was increased significantly (*p<*0.001), while catalase activity was found to be insignificantly increased (*p>*0.05) in the treated group as compared to the cadmium exposed group after 21 days treatment (Figures 1 and 2). The cadmium chloride exposed group showed a significant reduction (*p<*0.001) of glutathione reductase activity in plasma, brain and renal tissue as compared to the control group, while this enzyme was found insignificantly (*p>*0.05) decrease in liver tissue in comparison with the control group. After treatment with the drug for 21 days, this enzyme activity was significantly increased only in plasma while in all tissue homogenates this enzyme activity appeared insignificantly increased (*p>*0.05) in treated group as compared with the cadmium exposed group (Figure 3). GPx activity was significantly (*p<*0.001) decreased in plasma, hepatic and renal tissues of the cadmium exposed group as compared to the control group, while in the brain the enzyme activity was reduced insignificantly (*p>*0.05) as compared to the control group. After treatment with C+S with VRP1034 through intravenous route for 21 days, the enzyme activity was significantly elevated (*p<*0.001) in the liver and (*p<*0.05) in renal tissue while the activity was found insignificantly (*p>*0.05) increased in plasma and brain tissue of the treated group (Figure 4). The GSH level was significantly (*p<*0.001) reduced along with significantly increased GSSG level and their ratio in plasma and all tissues of the CdCl_2_ induced group was lowered after 21 days as compared to the control group. After treatment with the drug, these levels were increased along with significant increased their ratio in the plasma and tissues of treated group as compared to the cadmium exposed group (Tables 1 and 2).

**Figure 5:**
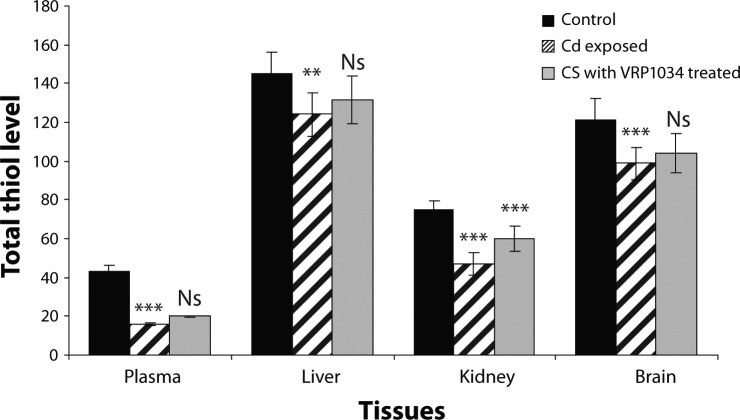
Effect of ceftriaxone plus sulbactam with VR1M 034 on Total thiol level in cadmium exposed rat. All data are Mean ± SD of each group. The level expressed in plasma (µmol/ml) whereas in tissues (µmol/g tissue). Data are compared between control *vs* Cd exposed group and Cd exposed *vs* C+S with VRP1034 treated group. Where ***p<0.001 (highly significant); **p<0.01 (significant); *p<0.05 (significant) and ^Ns^ *p>*0.05.

**Table 1:** Effect of ceftriaxone plus sulbactam with VRP1034 on GSH, GSSG levels in cadmium exposed group after 21 days of treatment.

Groups	Plasma		Brain		Liver		Kidney	
	GSH	GSSG	GSH	GSSG	GSH	GSSG	GSH	GSSG
Control group	3.96±0.96	0.81±0.17	4.89±0.88	0.55±0.11	5.78±0.55	0.64±0.06	2.60±0.04	0.54±0.13
Cadmium exposed group	1.55±0.48***	1.03±0.64^ns^	2.25±1.01***	0.89±0.091***	2.69±0.33***	0.91±0.05***	1.87±0.08***	0.71±0.15*
Ceftriaxone plus sulbactam with VRP1034 treated group	2.09±0.85^ns^	0.78±0.11^ns^	3.96±0.66***	0.64±0.10***	4.00±0.62***	0.77±0.08***	2.39±0.17***	0.60±0.09^ns^

All data are Mean ± SD of each group. The GSH and GSSG levels were expressed in the plasma (mg/dL) whereas in the tissues (µmol/g tissue). Newman-Keuls test was performed for statistical significance between control group vs Cd exposed group and Cd exposed group vs ceftriaxone plus sulbactam with VRP1034 treated group.

*** Where *p<*0.001 (highly significant)

** *p<*0.01(significant)

* *p<*0.05 (significant)

Ns *p>*0.05 (non significant).

**Table 2:** Effect of ceftriaxone plus sulbactam with VRP1034 on redox state and total thiol level in cadmium exposed group after 21 days of treatment.

Groups	Plasma		Brain		Liver		Kidney	
	GSH/GSSG	Total thiol	GSH/GSSG	Total thiol	GSH/GSSG	Total thiol	GSH/GSSG	Total thiol
Control group	4.88	4.33±0.98	8.89	121.4±10.85	9.03	145.36±10.88	4.81	75.12±4.52
Cadmium exposed group	1.5	1.57±0.34***	2.52	98.7±8.17***	2.97	124.21±11.20**	2.63	46.89±5.87***
Ceftriaxone plus sulbactam with VRP1034 treated group	2.68	1.99±0.73^ns^	6.19	104.3±10.21	5.19^ns^	131.66±12.55^ns^	3.98	60.24±6.54***

All data are Mean ± SD of eight animals of each group. The thiol level was expressed in the plasma (µmol/min/ml) whereas in the tissues (µmol/g tissue). Newman-Keuls test was performed for statistical significance between control group vs Cd exposed group and Cd exposed group vs ceftriaxone plus sulbactam with VRP1034 treated group.

*** Where *p<*0.001 (highly significant)

** *p<*0.01(significant)

* *p<*0.05 (significant)

Ns *p>*0.05 (non significant).

Total thiol level was significantly (*p<*0.001; *p<*0.001; *p<*0.01; *p<*0.001) lowered in plasma, brain, liver and renal tissue of the cadmium exposed group. Total thiol level was significantly elevated in plasma and tissues of the treated group after treatment with ceftriaxone plus sulbactam with VRP1034 for 21 days (Table 2). There were highly significant increases (*p<*0.001) in malondialdehyde (MDA) and myloperoxidase (MPO) levels in plasma and tissues of the CdCl_2_ exposed group as compared to the control group after 21 days. After treatment with ceftriaxone plus sulbactam with VRP1034, the MDA and MPO parameters were significantly lowered (*p<*0.001;*p<*0.05) in plasma, (*p<*0.01; *p>*0.05) in brain, (*p<*0.001) in liver and (*p<*0.001; *p>*0.05) in renal tissue of the treated group as compared to the cadmium chloride group (Table 3). Xanthine oxidase (XO) and glutathione-S transferase (GST) activities were significantly (*p<*0.001) altered in the plasma and tissues of the CdCl_2_ induced group as compared with the control group. The GST activity was significantly increased (*p<*0.001) along with significantly (*p<*0.001) reduced XO activity in plasma and tissues of the treated group as compared to the CdCl_2_ induced group after 21 days treatment. The GST and XO activities were found insignificant in renal and brain tissue in comparison to the cadmium exposed group (Table 4).

**Table 3:** Effect of ceftriaxone plus sulbactam with VRP1034 on oxidative stress parameters in cadmium exposed group after 21 days of treatment.

Groups	Plasma		Brain		Liver		Kidney	
	MDA	MPO	MDA	MPO	MDA	MPO	MDA	MPO
Control group	153.11±7.38	5.63±1.28	83.41±5.86	23.10±3.54	342.1±10.59	15.46±0.75	3.56±0.42	10.21±2.45
Cadmium exposed group	274.30±10.29***	8.44±0.98**	115.20±6.01***	32.45±2.09***	398.5±15.44***	40.22±1.39***	0.884±0.13***	17.50±1.12***
Ceftriaxone plus sulbactam with VRP1034 treated group	210.64±11.52***	6.25±1.99*	103.36±8.28**	29.42±2.63^ns^	361.87±8.56***	33.98±1.76***	1.96±0.22***	15.26±2.37^ns^

All data are Mean ± SD of each group. The MDA and MPO levels were expressed in the plasma (µmol/min/ml) whereas in the tissues (µmol/g tissue). Newman-Keuls test was performed for statistical significance between control group vs Cd exposed group and Cd exposed group vs ceftriaxone plus sulbactam with VRP1034 treated group.

*** Where *p<*0.001 (highly significant)

** *p<*0.01(significant)

* *p<*0.05 (significant)

Ns *p>*0.05 (non significant).

**Table 4:** Effect of ceftriaxone plus sulbactam with VRP1034 on xanthine oxidase (XO) and gluathione s transferase (GST)in cadmium exposed group after 21 days of treatment.

Groups	Plasma (nmol/min/ml)		Liver (nmole/min/g tissue)		Kidney (nmole/min/g tissue)		Brain (nmol/min/g tissue)	
	XO	GST	XO	GST	XO	GST	XO	GST
Control group	135.4±8.56	14.33±1.52	44.10±2.65	25.12±1.87	58.84±4.23	36.41±3.78	111.02±7.49	9.11±1.02
Cd exposed group	296.3±10.22***	18.96±1.10***	87.33±6.02***	51.05±2.64***	64.20±3.63*	42.16±2.81***	144.89±9.48***	13.40±1.47***
Ceftriaxone plus sulbactam with VRP1034 group	168.7±14.31***	13.74±0.55***	71.82±4.49***	37.0±2.09***	56.22±1.77***	40.33±1.59^ns^	139.45±8.80^ns^	10.00±1.12***

All data are Mean ± SD of each group. The XO and GST levels were expressed in the plasma (µmole/min/ml) whereas in the tissues (µmol/g tissue). Newman keuls test was performed for statistical significance between control group vs Cd exposed group and Cd exposed group vs ceftriaxone plus sulbactam with VRP1034 treated group.

*** Where *p<*0.001 (highly significant)

** *p<*0.01(significant)

* *p<*0.05 (significant)

Ns *p>*0.05 (non significant).

Hepatic parameters (SGOT, SGPT) were significantly increased in plasma and liver tissue while renal parameters (creatinine) was found to be increased in renal tissue of the cadmium exposed group as compared to the control group. These parameters were significantly lowered in plasma and tissues after treatment with ceftriaxone plus sulbactam with VRP1034 for 21 days (Table 5). Hematological parameters (Hb, RBC, WBC and HCT) were significantly altered in the cadmium exposed group as compared to the control group (Table 6). These parameters were improved after treatment with ceftriaxone plus sulbactam with VRP1034 for 21 days. The δ-ALAD enzyme activity was significantly inhibited in the blood of cadmium exposed group as compared to the control group. This activity was increased after treatment with ceftriaxone plus sulbactam with VRP1034 in the treated group as compared to the cadmium exposed group (Table 6). Cadmium concentration was found significantly (*p<*0.001) increased in blood and all tissues of the CdCl_2_ exposed group in comparison to the control group, while in brain tissue of the control group Cd concentration was not detected. The concentration of Cd was significantly (*p<*0.001) minimized in blood and all tissues in the group treated by intravenous route with ceftriaxone plus sulbactam with VRP1034 after 21 days. (Table 7). Depletion of zinc and Fe levels was observed in liver, brain and renal tissues, while the zinc level was increased in blood of the cadmium exposed group as compared to the control group. These levels were significantly increased in plasma and all tissues of group treated with ceftriaxone plus sulbactam with VRP1034 (Table 8).

**Table 5:** Effect of ceftriaxone plus sulbactam with VRP1034 on hepatic and renal parameters in cadmium exposed group after 21 days of treatment.

Groups	Plasma				Liver		Kidney
	SGOT	SGPT	Creatinine	ALP	SGOT	SGPT	Creatinine
Control group	10.10±2.45	12.97±2.63	1.02±0.12	19.56±3.54	20.48±0.85	8.63±1.21	0.86±0.053
Cadmium exposed group	88.56±4.56***	115.12±1.07***	7.54±1.75***	5.98±0.63***	73.63±1.55***	35.28±2.01***	5.84±0.13***
Ceftriaxone plus sulbactam with VRP1034 treated group	34.12±5.47***	66.39±3.25***	3.23±0.22***	14.25±1.14***	41.21±0.99***	18.56±2.84***	1.96±0.22***

All data are Mean ± SD of each group. These parameters were expressed in the plasma (IU/L) whereas in the tissues (mmol/g tissue). Newman-Keuls test was performed for statistical significance between control group vs Cd exposed group and Cd exposed group vs ceftriaxone plus sulbactam with VRP1034 treated group.

*** Where *p<*0.001 (highly significant)

** *p<*0.01(significant)

* *p<*0.05 (significant)

Ns *p>*0.05 (non significant).

**Table 6:** Effect of ceftriaxone plus sulbactam with VRP1034 on hematological and δ ALAD parameters in cadmium exposed group after 21 days of treatment.

Group	Hb (g/dL)	RBC (10^6^/µL)	HCT %	WBC (10^3^/µL)	δ-ALAD in blood (nmol/min/ml erythocyte)
Control	13.93±0.19	7.36±0.10	39.38±0.66	5.12±0.81	9.14±0.71
Cd-exposed	9.27±0.55***	7.01±0.08*	36.89±0.43**	11.25±1.31***	3.55±0.61***
Cd+Ceftriaxone plus sulbactam with VRP1034	11.22±0.99**	7.20±0.06^ns^	37.78±0.6*	7.84±0.58**	6.74±0.50***

All data are Mean ± SD of each group. Newman-Keuls test was performed for statistical significance between control group vs Cd exposed group and Cd exposed group vs ceftriaxone plus sulbactam with VRP1034 treated group.

*** Where *p<*0.001 (highly significant)

** *p<*0.01(significant)

* *p<*0.05 (significant)

Ns *p>*0.05 (non significant).

**Table 7:** Effect of ceftriaxone plus sulbactam with VRP1034 on cadmium level in blood and tissues after 21 days of treatment.

Groups	Blood (µg/ml)	Liver (µg/g tissue)	Kidney (µg/g tissue)	Brain (µg/g tissue)
Control group	0.15±0.011	0.68±0.13	0.12±0.01	ND
Cd exposed group	11.79±2.4***	16.87±1.17***	13.49±2.21***	3.89±0.27
Ceftriaxone plus sulbactam with VRP1034 treated group	6.75±0.89***	10.11±0.83***	9.55±1.07***	0.62±0.12***

All data are Mean ± SD of each group. Newman-Keuls test was performed for statistical significance between control group vs Cd exposed group and Cd exposed group vs ceftriaxone plus sulbactam with VRP1034 treated group.

*** Where *p<*0.001 (highly significant)

** *p<*0.01(significant)

* *p<*0.05 (significant)

Ns *p>*0.05 (non significant).

**Table 8:** Effect of ceftriaxone plus sulbactam with VRP1034 on Zinc and Fe levels in blood and tissues after 20 days of treatment.

Groups	Blood (µg/ml)		Liver (µg/g tissue)		Kidney (µg/g tissue)		Brain (µg/g tissue)	
	Zinc	Fe	Zinc	Fe	Zinc	Fe	Zinc	Fe
Control group	4.11±0.28	2.89±0.21	38.96±2.45	15.65±1.02	20.11±1.25	9.61±1.58	10.25±0.98	20.12
Cd exposed group	6.37±0.55***	0.33±0.014***	21.04±3.22***	8.87±1.56***	18.96±1.93^ns^	3.67±0.59***	6.69±0.71***	6.78±1.15***
Ceftriaxone plus sulbactam with VRP1034 group	5.02±0.19***	0.72±0.011***	24.56±1.5*	10.23±2.10^ns^	19.44±2.78^ns^	5.03±0.97*	9.85±1.28***	13.87±2.31***

All data are Mean ± SD of each group. Newman-Keuls test was performed for statistical significance between control group vs Cd exposed group and Cd exposed group vs ceftriaxone plus sulbactam with VRP1034 treated group.

*** Where *p<*0.001 (highly significant)

** *p<*0.01(significant)

* *p<*0.05 (significant)

Ns *p>*0.05 (non significant).

## Discussion

Cadmium from the environment enters into the body through the lungs and intestines. It is then transported into the blood and accumulates in the liver where it induces the synthesis of metallothionein (MT), a cytosolic protein to which cadmium binds. It is well known that heavy metals impair heme synthesis in the bone marrow and cause an increase of protoporphyrin-IX in erythrocytes. In the present study, there was significantly decreased δ-ALAD enzyme activity and hemoglobin levels along with increased cadmium concentration in blood and all tissues of the CdCl_2_ induced group as compared to the control group. Due to inhibition of δ-ALAD, the Hb level was reduced in the cadmium exposed group, which confirming that Cd interference with the heme synthesis pathway leads to anemia. The heme metabolic pathway is highly susceptible to alterations induced by metal ions. Kannan and Flora (2004) reported that hemoglobin level and δ-aminolevulinic acid dehydratase activity were inhibited during heavy metal exposure in rats. Cd induced oxidative stress by increased lipid peroxidation level and inhibited antioxidant enzymes required to prevent the oxidative stress cadmium toxicity (Kelley *et al.*, 1999). In our experiment, administration of CdCl_2_ for 21 days via gastric gavage resulted in a significantly elevated malonaldialdehyde level (a marker of free radical), myloperoxidase levels and XO (free radical generating enzyme) along with significant reduction of endogenous antioxidant enzymes activities (SOD, CAT, GR, GPx , total thiol, GSH) in plasma and tissues of the cadmium chloride induced group as compared to the control group. The level of GSSG and the ratio GSH/GSSG was also significantly reduced in the cadmium exposed group as compared to the control group after administration of CdCl_2_ for 21 days via gastric gavage. The depletion of GSH, increase in GSSG and the lowered GSH/GSSG ratio in blood, liver, brain and renal tissue were consistent with the accumulation of cadmium in these tissues. These alteration seem to be due to the generation of free radicals (Bray &Taylor, 1993). Our results were in accordance with various previous reports suggesting that cadmium metal can cause oxidative stress by interaction with -SH groups of the major intracellular defense glutathione. Other studies also reported increased lipid peroxidation in tissues of mice by a lowered tissue GSH level during cadmiun intoxication. Cd exposure decreases the endogenous antioxidant enzyme (SOD, catalase, GR and GPx) activities along with increased lipid peroxidation and MPO levels in plasma, liver, brain and renal tissue as compared to the control group. Similarly, there was a significant reduction of total thiol level in plasma and all tissues of the cadmium exposed group as compared to the control group. Total thiol level decreases in the cadmium exposed group were due to the binding of thiol with cadmium metal. Various studies reported that alterations in endogenous antioxidant enzyme activities in different organisms following cadmium exposure depended on different periods of time, the amount of cadmium and the affected organs (Tandon et al, 2003; Jurczuk *et al.*, 2006). Casalino *et al.*, (2002a, b) and Sen Gupta *et al.* (2004) reported similar results in experiments in rats. Asagba (2010) and Guilhermino *et al.* (1998) reported that cadmium exposure altered the hematological parameters in the cadmium exposed rat model. Thus in our study, there was a significant reduction in the hematological parameters (Hb, RBC and HCT%) in the cadmium exposed group. The hepatic and renal parameters were also significantly increased in the cadmium exposed group as compared with the control group. Renal impairment due to cadmium toxicity causes alterations in proximal tubules of kidney tissue. These findings also suggest that cadmium metal causes increased lipid peroxidation that imbalance the antioxidant defense system, which leads to organs damage and dysfunction. δ-ALAD activity decreased in blood along with significantly decreased Zn and Fe levels in blood and tissue of the cadmium exposed group as compared to the control group. The inhibition of δ-ALAD in the cadmium exposed group was due to interference with the heme synthesis pathway. The inhibition of micronutrient (Zinc, Fe) levels in the cadmium exposed group was due to cellular inhibitory action of these metals themselves.

After treatment with ceftriaxone plus sulbactam with VRP1034 for 21 days, these antioxidant enzyme activities, along with xanthine oxidase, lipid peroxidation, MPO levels, hepatic and renal parameters were significantly improved in plasma and tissues of the treated group as compared to the Cd exposed group. The GSH/GSSG ratio, δ-ALAD, cadmium concentration and micro nutrients levels were also improved in plasma and tissues of the treated group as compared to the Cd exposed group. The increased δ-ALAD, SOD, catalase, GR and GPx antioxidant enzyme activities and decreased lipid peroxidation, MPO and cadmium level in the blood and tissues indicate that ceftriaxone plus sulbactam with VRP1034 exert antioxidant and chelating properties. Ceftriaxone and sulbactam individually interact with heavy and trans metals and form a complex which chelates out from the sulfhydryl group of antibiotics. But due to the presence of VRP1034 in ceftriaxone plus sulbactam, this combination showed synergistic effect that enhanced the free radical scavenging and chelating properties. Various studies reported that ceftriaxone and sulbactam individually showed free radical scavenging properties (Lapenna *et al.*, 1995; Gunther *et al.*, 1993). Our previous findings also showed that ceftriaxone plus sulbactam with VRP1034 was an effective drug for removal of arsenic intoxication in rats (Dwivedi *et al.*, 2011). To our knowledge, there are no articles available on the combination of two antibiotics (betalactam and betalactamase inhibitor) exerting metal chelating properties. On the basis of the above results it is concluded that ceftriaxone plus sulbactam with VRP1034 is a protective and effective drug for removal of heavy metal from the body and plays a significant role in improving the endogenous antioxidant defense system along with reducing oxidative stress and protecting from hepatic and renal injury during cadmium toxicity.
